# Protection against Experimental Stroke by Ganglioside GM1 Is Associated with the Inhibition of Autophagy

**DOI:** 10.1371/journal.pone.0144219

**Published:** 2016-01-11

**Authors:** Li Li, Jinghua Tian, Mitchell King-Wei Long, Yong Chen, Jianfei Lu, Changman Zhou, Tianlong Wang

**Affiliations:** 1 Department of Anesthesiology, Xuanwu Hospital, Capital Medical University, Beijing, China; 2 Department of Anatomy, Heze Medical College, Shandong, China; 3 Osteopathic Medicine, Des Moines University, Des Moines, Iowa, United States of America; 4 Department of Neurology, People's hospital of Deyang City, Sichuan, China; 5 Department of Anatomy & Histology, School of Basic Medical Sciences, Peking University Health Science Center, Beijing, China; Indian Institute of Integrative Medicine, INDIA

## Abstract

Ganglioside GM1, which is particularly abundant in the central nervous system (CNS), is closely associated with the protection against several CNS disorders. However, controversial findings have been reported on the role of GM1 following ischemic stroke. In the present study, using a rat middle cerebral artery occlusion (MCAO) model, we investigated whether GM1 can protect against ischemic brain injury and whether it targets the autophagy pathway. GM1 was delivered to Sprague-Dawley male rats at 3 doses (25 mg/kg, 50 mg/kg, 100 mg/kg) by intraperitoneal injection soon after reperfusion and then once daily for 2 days. The same volume of saline was given as a control. Tat–Beclin-1, a specific autophagy inducer, was administered by intraperitoneal injection at 24 and 48 hours post-MCAO. Infarction volume, mortality and neurological function were assessed at 72 hours after ischemic insult. Immunofluorescence and Western blotting were performed to determine the expression of autophagy-related proteins P62, LC3 and Beclin-1 in the penumbra area. No significant changes in mortality and physiological variables (heart rate, blood glucose levels and arterial blood gases) were observed between the different groups. However, MCAO resulted in enhanced conversion of LC3-I into LC3-II, P62 degradation, high levels of Beclin-1, a large area infarction (26.3±3.6%) and serious neurobehavioral deficits. GM1 (50 mg/kg) treatment significantly reduced the autophagy activation, neurobehavioral dysfunctions, and infarction volume (from 26.3% to 19.5%) without causing significant adverse side effects. However, this biological function could be abolished by Tat–Beclin-1. In conclusion: GM1 demonstrated safe and robust neuroprotective effects that are associated with the inhibition of autophagy following experimental stroke.

## Introduction

Ischemic stroke is a consequence of vascular occlusion. The result is irreversible neural tissue injury, which is a major cause of death and disability worldwide. As the world’s population continues to age, the incidence of stroke is expected to grow, increasing the interest and need for novel approaches focused on improving the recovery and quality of life of stroke patients.

A key complication of ischemic stroke is neuron damage secondary to excitotoxicity, acute energy failure, or programmed cell death under ischemia/reperfusion conditions [1, 2]. Increasing evidence demonstrates that prolonged autophagy, a non-apoptotic route of type II programmed cell death, plays a role in cerebral ischemic injury amongst a variety of other neurologic conditions [3–5]. Autophagy is a programmed cell survival process that generally mediates breakdown and recycling of cellular components such as long-lived proteins and damaged organelles. However, persistent stress can promote high levels of autophagy resulting in cell death [6–8].

Massive activation of neuronal autophagy and its associated markers, LC3 and Beclin-1, have been firmly established in a variety of focal ischemic stroke models [5, 9]. Another notable factor includes the gangliosides. While GM2 and GM3 are induced transiently within the ipsilateral hemisphere after the induction of ischemic injury in the mouse [10], GM1, an important component of lipid rafts, can act as a neurotrophic factor [11–14]. GM1 has been shown to potentiate the action of neurotrophins and display a wide variety of central nervous system functions including promoting survival, differentiation[15], neurodegeneration [14, 16, 17], axon stability, and regeneration[18]. A plethora of studies have suggested that GM1 may be involved in the stroke process, specifically the orchestration of cell death and subsequent neurological dysfunctions [19]. However, the precise mechanism of action remains inconclusive.

In the present study, the role of GM1 in ischemic stroke and its potential regulation of neuronal autophagic activities were investigated. We hypothesized that GM1 could improve neurological outcomes via the inhibition of excessive autophagy in stroke development. Tat–Beclin-1 peptide was used to induce autophagy, and the effects of GM1 on infarction volume, mortality rate and neurological deficits were assessed. Additionally, the expression of P62, LC3, and Beclin-1 were measured as a potential mechanism of GM1 in conferring neuroprotective properties following ischemia-reperfusion injury.

## Materials and Methods

### Animals and treatments

All experimental procedures using rats in this study strictly followed recommendations provided by the National Institutes of Health Guide for the Care and Use of Laboratory Animals and were approved by the Bioethics Committee of Capital Medical University, Beijing, China. Sprague-Dawley male rats weighing 280~300 g were housed in a 12-hour light/dark cycle at a controlled temperature and humidity with free access to food and water. All surgery was performed under chloral hydrate anesthesia, which was specifically reviewed and approved by the ethics committee, and all efforts were made to minimize suffering. During surgery, rats were anesthetized with chloral hydrate (0.4 g/kg, i.p.), which was selected because, unlike alternative anesthetics, it has not been shown to upregulate autophagy [20]. Then rats were subjected to focal cerebral ischemia by intraluminal middle cerebral artery occlusion (MCAO) with a nylon suture as previously described [21, 22]. After 2 hours of MCAO, the suture was withdrawn to allow reperfusion. The heart rate, blood glucose levels, and blood gases were monitored before, during, and after ischemia. Core body temperature was maintained at 37°C. Sham-operated rats underwent a similar procedure with the exception of nylon suture occlusion and reperfusion. Ten rats that died during the operation under anesthesia were not included.

GM1 (purity>98%, Santa Cruz Biotechnology, Inc., CA, USA, sc-202624A) was delivered at 3 doses (25 mg, 50 mg, 100 mg in 10 ml saline/kg) by intraperitoneal injection soon after reperfusion and then once daily for 2 days. The same volume of saline was administered as a control. Tat–Beclin-1 (Millipore, Billerica, MA, USA, 506048#, 15 mg in 10 ml saline/kg, i.p.) was given at 24 and 48 hours post-MCAO. Animals were randomly assigned to the following five groups: sham surgery (n = 17), MCAO+saline (n = 27), MCAO+GM1 (n = 33 in the 50 mg/kg group, n = 14 in the 25 mg/kg group and n = 14 in the 100 mg/kg group), MCAO+Tat–Beclin-1 (n = 14), and MCAO+Tat–Beclin-1+GM1 (n = 13).

Rats were monitoring hourly for the first 4 hours after the MCAO operation. The rats were then observed twice daily for the next 2 days, once in the morning and once in the afternoon. Endpoints used included heart rate, blood pressure, and temperature. Post-operative care consisted of monitoring heart rates, use of a heating pad to alleviate cold temperatures, oxygen therapy for poor ventilation, closely monitoring the incision site for hemorrhage and infection, and administration of pain killers in response to characteristic changes in behavior and/or dietary habits. Fifteen rats died post-operatively. The surviving rats were neurologically tested by a masked investigator and euthanized 72 hours after MCAO. The brains were removed for the preparation of slices or lysates.

### TTC Staining

The infarct size of the ischemic cortex was measured as described previously [23]. In brief, 2-mm thick coronal sections were dissected using a rat brain slicer (Matrix, ASI Instruments, Houston, TX, USA) 72 hours after MCAO. The slices were incubated in 2% 2,3,5-triphenyltetrazolium chloride (TTC) solution (Sigma-Aldrich Chemical Co., St. Louis, MO, USA) at 37°C for 30 minutes, and fixed with 4% paraformaldehyde in PBS (pH 7.4) at 4°C for 6 hours. Normal tissue stains red while infarcted tissue with absent mitochondrial enzyme activity fails to stain and appears white. The infarct volume of each section was traced and measured by manually outlining the margins of non-ischemic areas using an image analysis system (Imaging-Pro-Plus, Silver Spring, MD, USA). To account for the increase in brain volume secondary to post-ischemic brain edema, infarct size was normalized to the contralateral cortex and expressed as a percentage according to the following formula:

$$\text{Infart Volume}=\frac{\text{Left Hemisphere Volume}\;-\;\text{Right Nonischemic Volume}}{\text{Left Hemisphere Volume}}\text{x}\;100$$

### Mortality and Neurobehavioral Deficits

Mortality was calculated at 72 hours after MCAO. Neurobehavioral deficits were assessed and scored based on the scoring system of Garcia et al in a blinded fashion [24].

### Immunofluorescence Staining

Animals were deeply anesthetized with chloral hydrate and perfused transcardially with saline followed by 250ml cold 4% paraformaldehyde (PFA) in 0.1M phosphate buffer (PB; pH 7.4). Brains were dissected, post-fixed in PFA for 12~18 hours, and transferred to 30% sucrose in 0.1 M PB for at least 48 hours at 4°C. Serial sections of the entire brain were cut at 10μm thickness on a cryostat (Leica CM3050S). Double fluorescence labeling was performed as described previously [25]. In brief, sections were rinsed 3 times in 0.01M PBS for 5 min each, permeabilized with 0.3% Triton X-100 in PBS for 30 min at 37°C, blocked with 1% BSA in PBS for 2 hours at room temperature, and incubated with the following primary antibodies: mouse anti-Beclin-1, and rabbit anti-LC3-II (Santa Cruz Biotechnology, Inc., CA, USA) at a concentration of 1:50~1:200 diluted in 0.01M PBS in a humidified chamber overnight at 4°C. Sections were then washed with 0.01M PBS and incubated for 2 hours with the secondary antibodies (anti-mouse IgG labeled with Alexa Fluor-488, and anti-rabbit IgG labeled with Alexa Fluor-568, 1:200, Jackson ImmunoResearch Laboratories, Inc., West Grove, PA, USA) at room temperature. Tissue sections were washed 3 times in 0.01M PBS for 5 min each, and then incubated with 2μg/ml Hoechst 33258 (Sigma Aldrich, Inc., St-Louis, MO, USA) for 10 min at room temperature to contain nuclei. Primary antibody incubation was omitted in some sections as a negative control. Images were acquired using an OLYMPUS BX51 microscope.

### Western Blotting

Animals were sacrificed for tissue harvest at 72 hours after MCAO. Tissue from the brain cortex, including the penumbra area, were dissected and immediately frozen in liquid nitrogen and stored at -80°C. Samples were homogenized in RIPA buffer (Santa Cruz) with protease inhibitor cocktail (Sigma) and incubated on ice for 30min. Lysates were centrifuged at 14000 x g for 25min at 4°C. Total protein concentrations were then measured using the Bradford assay (Santa Cruz). Supernatants were mixed with an equal volume of 2x Laemmli buffer (Santa Cruz) and denatured at 95°C for 5min. Protein samples (50 μg) were loaded on polyacrylamide gels, electrophoresed, and transferred to 0.45μm nitrocellulose membranes (Bio-Rad). The membranes were then blocked with 5% milk (Sigma) in 1xTBST for 2 hours at room temperature, followed by incubation with the primary antibodies (mouse anti-P62, rabbit anti- LC3-II/ LC3-I or mouse anti-Beclin-1, Santa Cruz) at 1:1000 dilution in 3% milk overnight at 4°C. The membranes were then washed 3x5min in 1xTBST and probed with the secondary antibodies (goat anti-rabbit IgG, goat anti-mouse IgG, Santa Cruz, 1:8000) for 1 hour at room temperature. Immunoblots were then washed and probed with an ECL Plus chemiluminescence reagent kit (Amersham Biosciences, Arlington Heights, IL, USA) for 5min and detected by exposure to Kodak X-ray film. The optical density of the bands was quantified by Image J software. β-Actin monoclonal antibody (goat anti-β-Actin, Santa Cruz, 1:5000) was also probed, and used as a sample loading control for normalization, and the data were expressed as the ratio to β-Actin.

### Data Analysis

The analysis of the data was performed using SigmaPlot software. Data are expressed as the mean ± standard error of the mean and analyzed with one-way analysis of variance (ANOVA) followed by the Student-Newman-Keuls method. The neurobehavior scores were analyzed with Kruskal–Wallis one-way ANOVA followed by multiple comparison procedures by the Dunn method. Data are expressed as the median ± 25th-75th percentiles. Statistical significance was defined as *P*< 0.05.

## Results

### GM1 Does Not Significantly Affect Mortality or Physiological Variables of MCAO Rats

**Fig 1:**
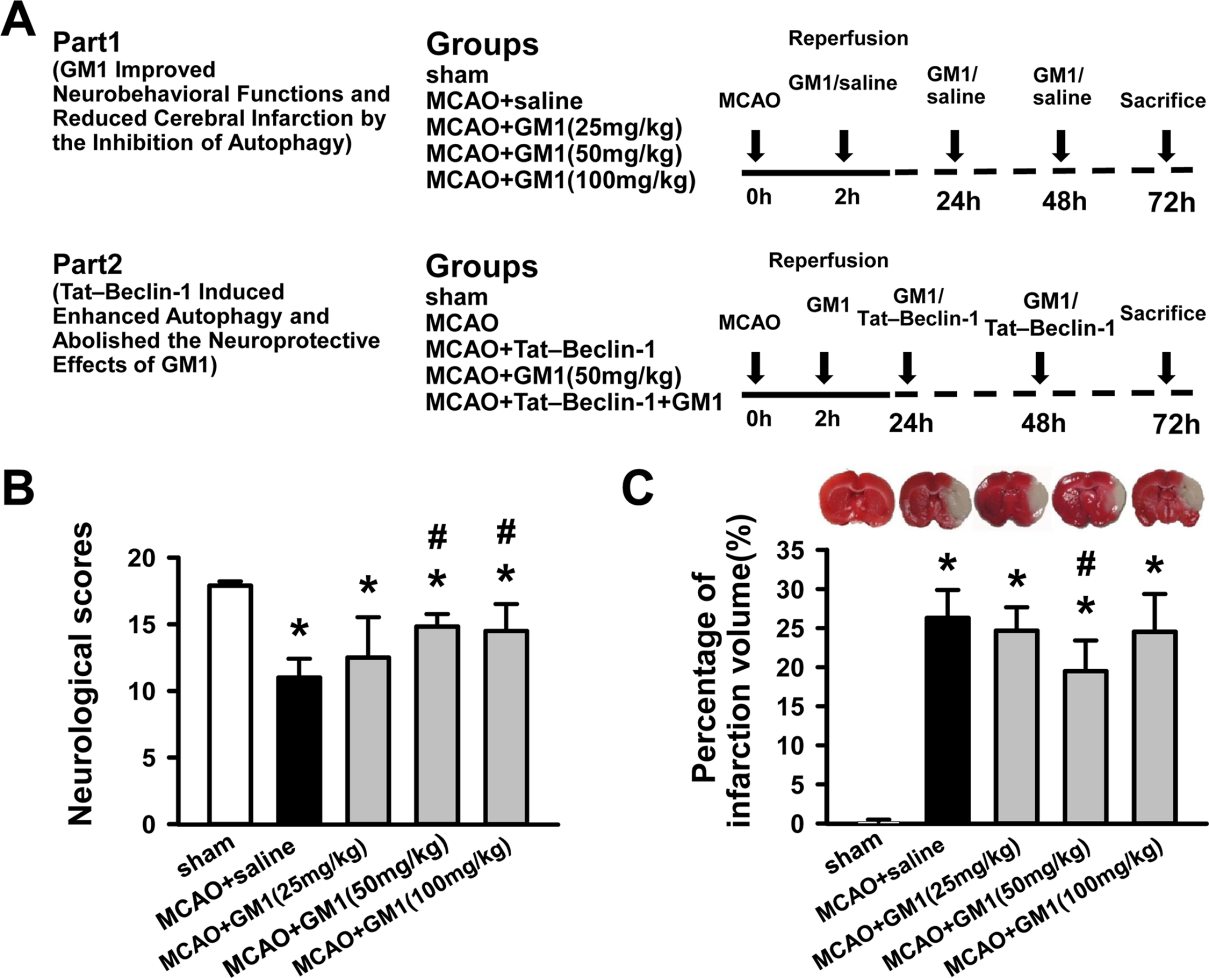
GM1 (50 mg/kg) treatment improves neurological scores and reduces infarct volume at 72 hours after MCAO. GM1 was administrated soon after reperfusion and then once daily for 2 days at three doses (25, 50, and 100 mg/kg) by intraperitoneal injection. MCAO rats were injected with the same volume of saline as a control. (A) Experimental design and animal treatment. (B) Neurological scores for the modified Garcia test in sham, MCAO+saline and GM1 (25, 50, 100 mg/kg) treatment groups. n = 10–12 rats per group. (C) Representative images of TTC stained sections and infarct volume in sham, MCAO+saline and GM1 treatment groups. n = 6 per group. *p < 0.05 vs sham; #*p* < 0.05 vs MCAO+saline.

To investigate the potential for GM1 to protect against ischemic brain injury and whether this protection involves the autophagy pathway, we employed a rat MCAO model. The experimental design and animal treatment are shown in Fig 1A. The study involved two parts: In Part 1, the protective effects of GM1 were assessed for three different doses of GM1; and in Part 2, the ability of Tat-Beclin-1 to attenuate the protective effects of 50 mg/kg GM1 was assessed. No sham-operated animal died in the study (0/17 rats). There was mortality observed in all of the MCAO groups, but the amount of mortality was not significantly different among the groups (14.8% [4/27 rats] in the MCAO+saline group, 14.2% [2/14 rats] in the MCAO+25 mg/kg GM1 group, 12.1% [4/33 rats] in the MCAO+50 mg/kg GM1 group, 14.2% [2/14 rats] in the MCAO+100 mg/kg GM1 group, 14.2% [2/14 rats] in the MCAO+Tat–Beclin-1 group and 7.7% [1/13 rats] in the MCAO+Tat–Beclin-1+GM1 group [*P*>0.05]).

Additionally, no significant changes of physiological variables (heart rate, blood glucose levels, blood gases analysis [pH, pCO_2_, pO_2_, Na^+^, Ca^2+^, Cl^-^, BUN, Glu, Hct, HB, BE, tCO_2_, BB, SBE, SBC, HCO3^-^, sO_2_], and body weight) were observed between the different groups throughout the study (data not shown).

### GM1 (50 mg/kg) Improves Neurobehavioral Functions and Reduces Cerebral Infarction

To evaluate the sensorimotor deficits after MCAO, a modified Garcia test was performed at 72 hours following MCAO (Fig 1B). No deficits were observed in sham animals. In contrast, a significant decline of neurological scores was detected in the MCAO +saline group (*P*<0.05 vs. sham). After both the medium-dose (50 mg/kg) and high-dose (100 mg/kg) GM1 treatment, there was a statistically significant improvement in neurobehavioral function when compared to MCAO +saline group (*P*<0.05).

The cerebral infarction at 72 hours after MCAO was also evaluated using TTC staining. Representative samples of TTC-stained brain sections with corresponding infarction volumes are shown in Fig 1C. The infarction volume revealed a marked increase in the MCAO group compared to the sham animals (26.3±3.6% vs. 0.2±0.2%, *P*<0.05). However, the infarct ratios were decreased from 26.3% to 19.5% in rats with the medium-dose GM1 (50 mg/kg) treatment (*P*<0.05 vs. MCAO). Although the brain infarction showed a trend toward reduction following low-dose (25 mg/kg) and high-dose (100 mg/kg) administration, there was no statistical significance reached (25 mg/kg, 24.7±3.0%, 100 mg/kg 24.6±4.8% vs. MCAO 26.3±3.6%, *P*>0.05). Overall, our results demonstrate that injection of GM1 (50 mg/kg) improves neurobehavioral functions and reduces infarct volume after ischemic insult despite having no effect on the mortality rate in the MCAO model. Based on these findings, a dose of 50 mg/kg of GM1 was used in subsequent studies.

### GM1 Decreases LC3-II and Beclin-1 Levels and Increases P62 Levels Following MCAO Injury

**Fig 2:**
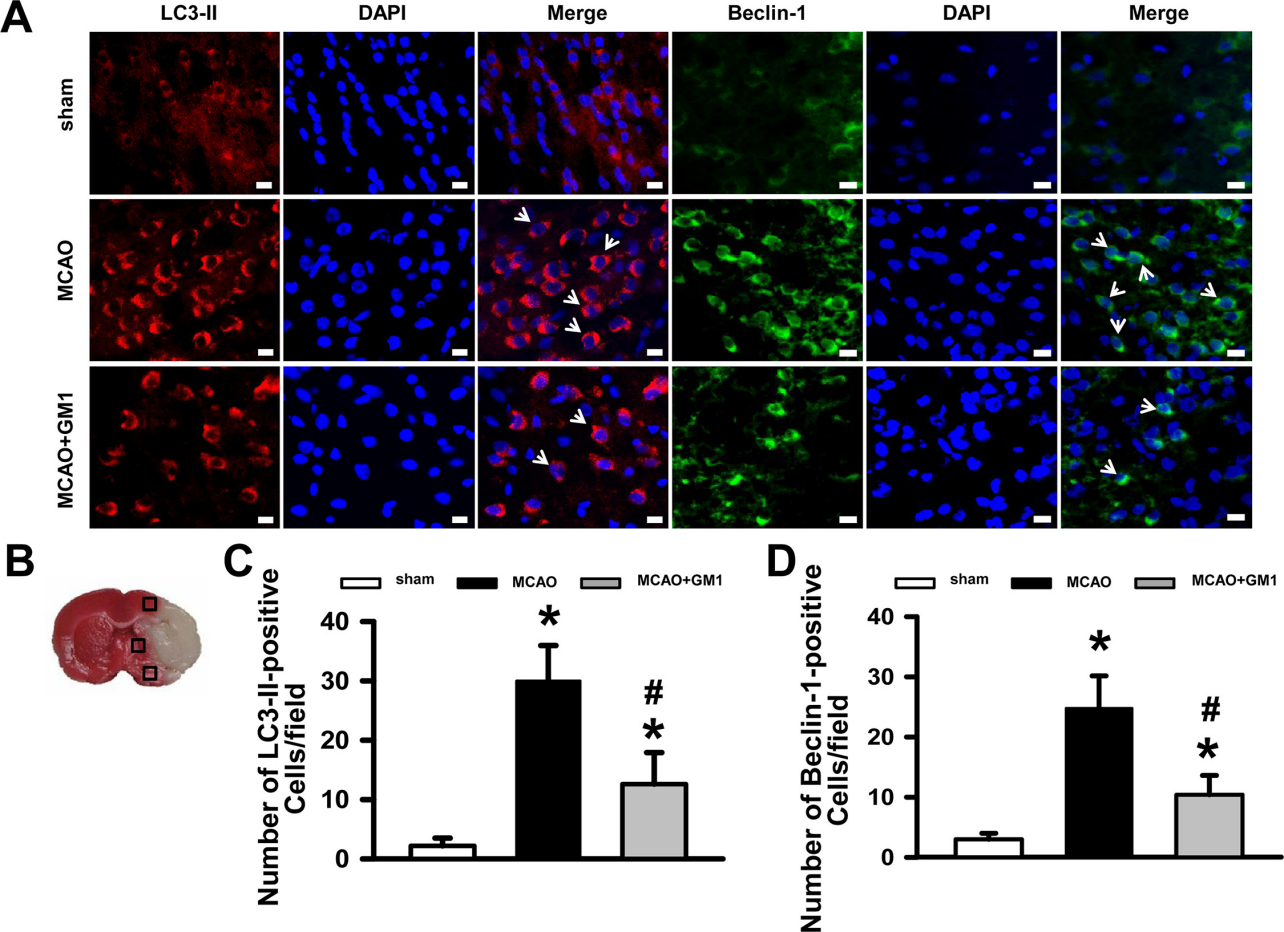
Immunofluorescence staining for LC3-II and Beclin-1 expression in sham, MCAO+saline and GM1 (50 mg/kg) treatment groups 72 hours following MCAO injury. (A) Representative images of LC3-II (red) and Beclin-1 (green) staining in the perihematomal area. Bar = 50μm. (B) Schematic diagram showing examples of the areas (black squares) that were selected for counting of LC3-II and Beclin-1 positive cells in the perihematomal region. (C) Quantification of LC3-II positive cells in the perihematomal region (10 fields/brain). (D) Quantification of Beclin-1 positive cells in the perihematomal region (10 fields/brain). The data show that GM1 treatment significantly reduced the number of LC3-II and Beclin-1 positive cells after MCAO insult. n = 5 per group. *p < 0.05 vs sham; #*p* < 0.05 vs MCAO.

MAP1LC3B/LC3 and SQSTM1/p62 are most frequently used as markers to measure autophagy flux. LC3, the microtubule-associated protein light chain 3, exists in a cytosolic form (LC3-I) and an autophagosome-associated membrane-bound form (LC3-II). The ratio of conversion from LC3-I to LC3-II is closely correlated with the extent of autophagosome formation [26]. P62, a selective autophagy substrate, was originally discovered as a scaffold in signaling pathways regulating cell growth and proliferation; however, it was also determined to bind to several autophagy substrates, such as ubiquitinated proteins, damaged mitochondria and signaling molecules, promoting their autophagic clearance[27]. Beclin-1, another key factor in the autophagic process, is essential for the recruitment of other autophagic proteins during the expansion of the pre-autophagosomal membrane [28, 29]. To determine the effect of GM1 on neuronal autophagic activities after MCAO, we first measured the LC3-II and Beclin-1 expression levels in brain tissue by immunostaining 72 hours after brain ischemia and quantified both the LC3-II and the Beclin-1 positive cells in the penumbra area (Fig 2). The results demonstrate that continuous treatment with GM1 caused a significant reduction in the number of both LC3-II positive cells (12.6±4.8 vs. MCAO 30±5.3, *P*<0.05; Fig 2C) and Beclin-1-positive cells (10.4±2.9 vs. MCAO 24.8±4.8, *P*<0.05; Fig 2D).

**Fig 3:**
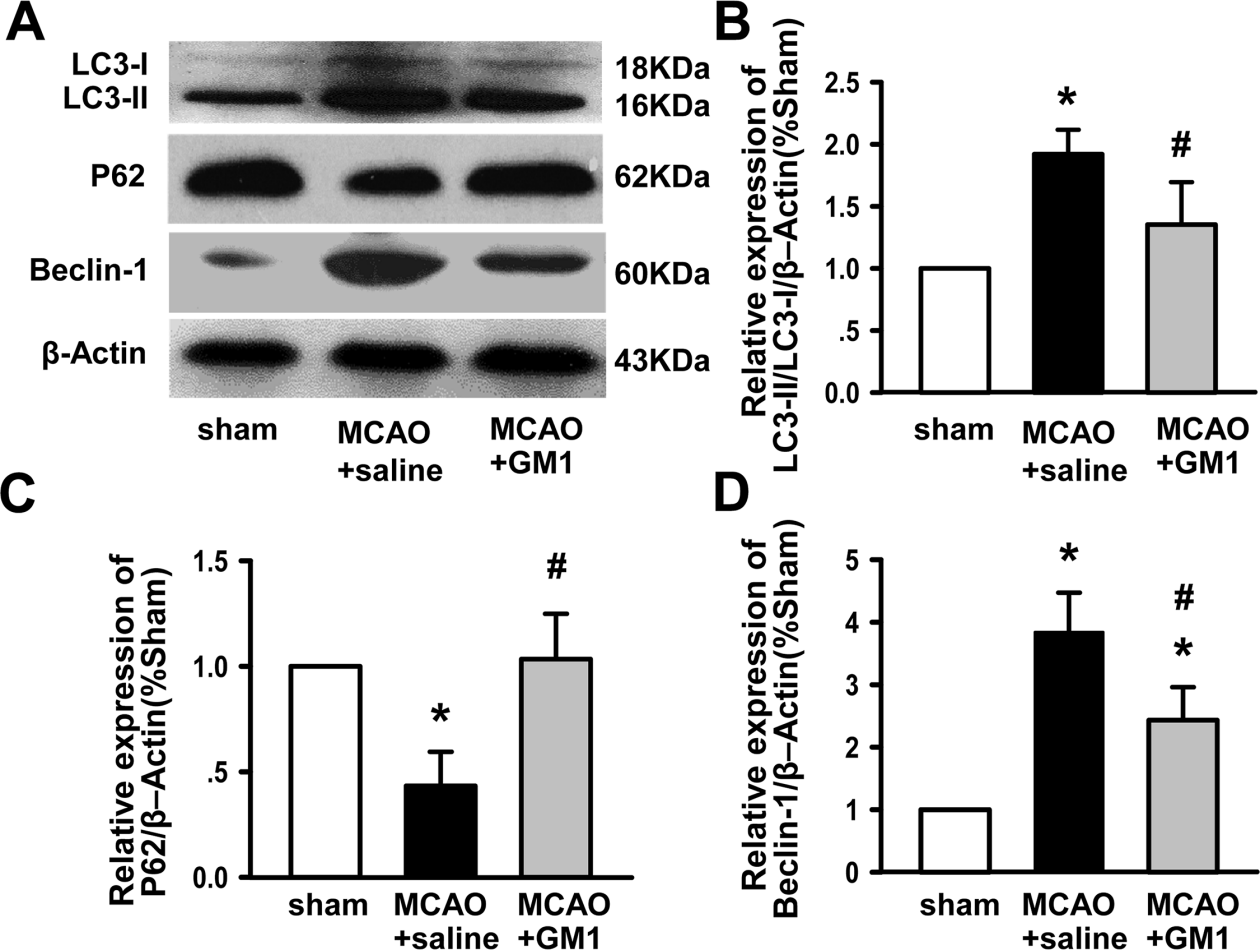
GM1 inhibits neuronal autophagic activity following MCAO injury. (A) Representative Western blotting images of LC3-I, LC3-II, P62, and Beclin-1 in the ipsilateral hemisphere at 72 hours after MCAO injury. β-Actin is shown as a loading control. (B) Quantification of the LC3-II/LC3-I ratio in the ipsilateral hemisphere at 72 hours after MCAO injury. (C) Quantification of P62 in the ipsilateral hemisphere at 72 hours after MCAO injury. (D) Quantification of Beclin-1 in the ipsilateral hemisphere at 72 hours after MCAO injury. n = 6 per group. *p < 0.05 vs sham; #*p* < 0.05 vs MCAO+saline.

To verify these findings, we performed Western Blotting analysis of LC3, Beclin-1 and P62 (Fig 3). MCAO promoted a significant increase in LC3-II and Beclin-1 levels (*P*<0.05 vs. sham), which was obviously suppressed by subsequent GM1 injection (*P*<0.05 vs. MCAO), though there was no significant difference in LC3-I levels between sham and MCAO or GM1 rats (Fig 3B and 3D). Conversely, the expression of P62 was significantly decreased following MCAO and markedly increased with GM1 treatment compared to MCAO rats (*P*<0.05, Fig 3C). These findings confirm the role of GM1 in attenuating the effects of MCAO in rats.

### Tat–Beclin-1 Enhances Autophagy Levels and Abolishes the Neuroprotective Effects of GM1

**Fig 4:**
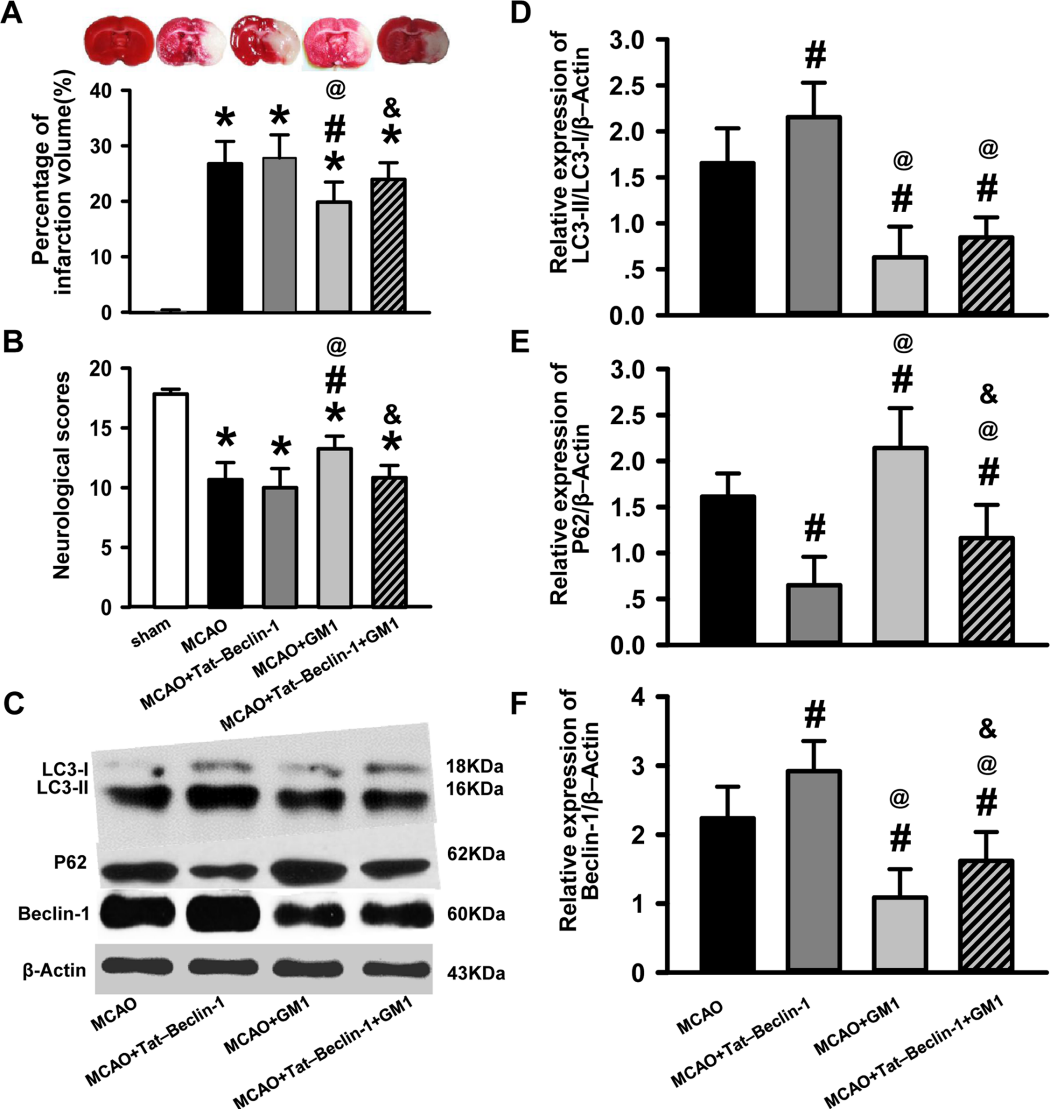
Enhanced autophagy induced by Tat–Beclin-1 abolishes the neuroprotective effects of GM1. (A) Representative images of TTC stained sections and quantification of infarct size in sham, MCAO+saline, MCAO+Tat–Beclin-1, MCAO+GM1, and MCAO+Tat–Beclin-1+GM1 groups. n = 6 per group. (B) Neurological scores for the modified Garcia test. n = 6–12 per group. (C) Representative Western blotting images of LC3-I, LC3-II, P62, Beclin-1 and β-Actin in MCAO+saline, MCAO+GM1, MCAO+Tat–Beclin-1, and MCAO+Tat–Beclin-1+GM1 groups. n = 6 per group. (D) Quantification of LC3-II/LC3-I in the ipsilateral hemisphere. (E) Quantification of P62 in the ipsilateral hemisphere. (F) Quantification of Beclin-1 in the ipsilateral hemisphere.*p < 0.05 vs sham; #*p* < 0.05 vs MCAO+saline; @*p* < 0.05 vs MCAO+Tat–Beclin-1; & *p* < 0.05 vs MCAO+GM1.

To test whether autophagy is involved in the neuroprotective function of GM1, Tat–Beclin-1 was used to enhance autophagy post-MCAO. As expected, Beclin-1 levels were significantly increased in the MCAO+Tat-Beclin-1 rats as compared to the MCAO rats. Furthermore, Tat–Beclin-1 application resulted in an enhanced conversion of LC-1 into LC3-II and enhanced P62 degradation, confirming its role as an autophagy enhancer. Conversely, the administration of both Beclin-1 and GM1 led to a reduction in the levels of Beclin-1 expression, LC3 conversion, and P62 degradation as compared to Tat-Beclin-1 alone, suggesting that GM1 can reverse the effects of Beclin-1 on autophagy (*P*<0.05, Fig 4A–4F). Tat-Beclin-1 did not clearly increase the infarct volume nor worsen the neurobehavioral functions compared to MCAO rats; however, the effects of GM1 on cerebral infarction reduction and neurobehavioral improvement were abolished by Tat-Beclin-1(*P*<0.05, Fig 4A and 4B). These findings further suggest that modulation of the levels of autophagy by GM1 contributes to its ability to attenuate the effects of MCAO.

## Discussion

Nearly 15 million people worldwide suffer from stroke each year, and this number continues to grow as a result of the aging population. The ensuing symptoms are a burden to the victims, their families, and the surrounding community (WHO The Atlas of heart disease and stroke section 15: Global burden of stroke). Despite the continued efforts to develop new pharmacological strategies, there are no effective treatment options as of yet. In the present study, to explore the potential activity and mechanism of GM1 in providing protection against ischemic brain injury, we investigated the effects of GM1 on MCAO-induced brain injury.

GM1 has been a focus of research investigations due to its function as a neurotrophic drug and its ability to penetrate the blood-brain barrier [30]. Although the development of the acute inflammatory polyneuropathy Guillain-Barre´ syndrome (GBS) following intravenous ganglioside treatment resulted in the withdrawal of GM1 from European market [31], this adverse effect was shown to be rare, and the relationship between exogenous gangliosides and GBS remains controversial [32, 33]. These drugs are still available and have been extensively prescribed in other markets including China, where a multitude of neurological maladies have been treated with gangliosides in the absence of resultant GBS or other severe adverse events [34–37]. In a trail conducted by Schneider *et al*., a 5-year clinical course confirmed the long-term safety of GM1 therapy and suggested favorable efficacy for Parkinson's disease patients [35]. Furthermore, the safety and efficacy of GM1in treating ischemic stroke has also been suggested in numerous clinical trials [38–40]. To understand mechanisms of GM1 that contributes to its therapeutic potential, the present study sought to evaluate its effects in a stroke animal model and its dose-dependent effects on neurological improvement. We did not observe any statistically significant differences in mortality between groups. However, the medium dose (50 mg/kg) GM1 treatment significantly improved neurological performance and alleviated cerebral infarction, while the low dose (25 mg/kg) and high dose (100 mg/kg) treatments did not. The precise mechanisms responsible for the neuroprotective effects of GM1 remain uncertain, but, as demonstrated by our study, GM1 may act in part through the regulation of neuronal autophagic activity. Autophagy (self-eating) is generally viewed as a cell survival mechanism in response to various stress conditions, and occurs without typical hallmarks of apoptosis. On the other hand, enhanced autophagy can also mediate cell death in cerebral ischemia [41]. Brain neuronal death following neonatal hypoxia/ischemia injury is largely prevented by Atg7 deficiency, which is essential for autophagy [42], whereas, promotion of autophagy with rapamycin augments cell death in insulin-deficient mice in adult hippocampal neural stem cells [43]. In support of a role for GM1 in regulating autophagy, Batten disease (juvenile neuronal ceroid lipofuscinosis) is associated with reduced autophagy, as well as enhanced level of GM1 [44]. In the present study, we demonstrated that MCAO resulted in a significant increase in LC3-II and Beclin-1 levels, which were markedly reduced by early initiation of GM1 (50 mg/kg) at 72 hours after reperfusion. Additionally, immunofluorescence staining showed a dramatic increase in the number of LC3-II and Beclin-1 positive cells post-MCAO, both of which were significantly reduced in the penumbra area after GM1 administration when compared with saline-treated rats. These findings suggest that autophagy might be involved in the neuroprotective function of GM1.

To further test whether the neuroprotective effects of GM1 might be explained in part by its ability to modulate autophagy, we tested whether Tat–Beclin-1, a specific cell-permeable autophagy-inducing peptide, can attenuate the effects of GM1. Tat–Beclin-1 was recently identified by Shoji-Kawata and colleagues by domain mapping of the autophagy protein Beclin-1[45]. It has been shown to efficiently induce autophagy in vivo and in vitro, decrease the small polyglutamine expansion protein aggregates, and reduce mortality in chikungunya or West Nile virus-infected mice by interacting with HIV-1 Nef [45, 46]. Consistent with these studies, in the present study, injection with Tat–Beclin-1 resulted in enhanced conversion of LC3-1 into LC3-II and P62 degradation. Furthermore, GM1 mediated cerebral infarction reduction was blocked by Tat–Beclin-1. Thus, these findings support the hypothesis that GM1 may exert its neuroprotective effects in ischemic stroke, in part, by regulating neuronal autophagic activity.

Our results show that the neuroprotective effects of GM1 only can be observed within a narrow window of concentration, and that GM1 has no statistical neuroprotective ability when both MCAO and Tat-Beclin-1 are applied, which is suggestive of a threshold of autophagy above which neurotrophic activity cannot be observed within our model. Furthermore, it is likely that other activities, in addition to autophagy, can contribute to the function of GM1. The current study has the following limitations: 1) only one autophagy inducer was included and no autophagy inhibitor was studied; 2) only short-term (3d post-stroke) effects were observed, whereas longer term (14d, 21d post-stroke) effects were not studied; 3) while the Garcia test was performed to assess neurobehavioral activity, tests for learning, memory, and social interaction were not performed. For this reason, further experimentation, including additional dosing experiments, specific autophagy inhibitor interventions, long-term studies and additional behavior studies, will be critical for determining the potential of GM1 in clinical ischemia. Nevertheless, the present results suggest that the early use of GM1 is safe and effective in rats after ischemic brain injury, and describe a new mechanism that could account for the neuroprotective effects of GM1.

## Supporting Information

S1 File The ARRIVE Guidelines Checklist. Animal Research: Reporting In Vivo Experiments.
(PDF)
